# Alcohol consumption and sports-betting among young male motorcycle taxi *boda boda* riders in urban southwestern Uganda

**DOI:** 10.1186/s12889-021-10406-7

**Published:** 2021-02-16

**Authors:** Stella Cheryl Nabifo, Jonathan Izudi, Francis Bajunirwe

**Affiliations:** Department of Community Health, Mbarara University of Science and Technology, P.O.BOX 1410 Mbarara, Uganda

**Keywords:** Sports-betting, Alcohol consumption, Uganda, Young males

## Abstract

**Background:**

The popularity of sports-betting is growing globally and may be associated with alcohol consumption among young adults. In this study, we examine the relationship between alcohol consumption plus other substances with sports-betting in a group of young adult males in Uganda.

**Methods:**

We conducted a cross-sectional study and interviewed male motorcycle taxi *boda boda* riders in the southwestern Uganda town of Mbarara. We asked questions about alcohol consumption, smoking plus history and frequency of sports-betting.

**Results:**

We enrolled 401 riders. The median age was 29.0 years, interquartile range 25–32 years. Seventy four (18.5%) had engaged in sports-betting in the past 30 days. Among those that reported sports-betting, 16(21.6%) engaged daily or almost daily. Alcohol use was significantly associated with sports-betting with an adjusted risk ratio (aRR) of 2.08(95% CI 1.36, 3.18) among moderate drinkers but not among heavy drinkers in comparison to low or non-drinkers. Cigarette smoking was significantly associated with sports-betting with an aRR 1.85(95% CI 1.13, 3.01).

**Conclusion:**

Sports-betting is common among these young male motorcycle riders, and is associated with both alcohol consumption and cigarette smoking. Interventions to regulate sports-betting may be co-packaged with those to reduce alcohol consumption and cigarette smoking among young adult males.

**Supplementary Information:**

The online version contains supplementary material available at 10.1186/s12889-021-10406-7.

## Background

There is a global increase in the popularity of sports-betting and this is likely due to the involvement of sports-betting companies in sponsorship and advertisement in popular sports events such as soccer [1]. The number of sports-betting companies involved in shirt sponsorship for football in European leagues has grown exponentially. In the top five European soccer leagues namely England, Germany, Italy, France, and Portugal, shirt sponsorship grew from one deal in the 2002/3 season to 26 in the 2010/11 season [2]. In the widely popular English Premier League alone, gambling companies that have first team shirt sponsorships grew from four deals in 2008, to six in 2012, and ten in 2017, accounting for half of the 20 English Premier League teams [3].

Soccer is the most popular game in majority of countries in sub Saharan Africa. The European soccer leagues are extremely popular in Africa and therefore soccer sponsors such as sports betting companies are likely to reach a very wide audience on the continent through their advertisement. Sports betting in Uganda is legal and one of the rapidly expanding industries now with presence of international betting companies throughout the country [4]. The betting industry has grown over the years that by June 2015, there were at least 23 licensed betting companies that were operational, with at least 1000 betting outlets in the country [5]. The companies have made betting even more accessible through the provision of online services that customers can access through their mobile phones and telephone service providers [6]. The sports betting industry is well-regulated and government has instituted regulations that only individuals who have reached the legal age of ≥18 years are permitted to participate [6].

Existing data suggest that males are more knowledgeable and engage more in sports betting and those that have peers who gamble tend to generally have positive attitudes towards sports betting [7]. In Uganda, a previous study reports that younger people are more likely to engage in sports betting and do this both for fun and to win money [8]. The same young crowd of males is also drawn to soccer matches where they are ardent supporters of the mostly foreign soccer leagues in Europe. The linkage between soccer and sports-betting or gambling advertisement exposes the football fans to the allure of sports-betting and poses potential public health threats to the soccer fans, majority of who are minors or young adults, because of the potential risk of developing problem gambling [9–11] and addiction. Gambling also increases the risk for financial problems, mental health conditions such as anxiety, depression, suicide, and problematic relationships at the family level particularly with spouse, children, and parents [12]..

The global marketing of sports-betting and alcoholic products together in live sports events such as soccer matches is very common [13] and betting advertisement associates significant moments of sports such as scoring goals with alcohol [14]. Alcohol consumption has been linked to gambling including sports-betting. Several studies have confirmed the association between alcohol consumption and sports-betting [15, 16] and also with smoking or substance abuse [17]. However, most of these studies have been conducted in resource rich settings.

In resource limited settings like Uganda, because of the popularity of soccer, exposure to sports-betting advertisement is increasing and anecdotal reports suggest that sports-betting may also be on the rise especially among youth in urban and peri-urban areas. Motorcycle taxis commonly referred to as *bodabodas* are operated by a predominantly youthful urban male population, who earn a daily income and may be a soft target for the sports-betting industry. There is limited empiric data on the frequency of gambling in the populations that have been targeted by the sports-betting companies.

Therefore, the purpose of this study was to establish the frequency and pattern of sports-betting, and the relationship between alcohol consumption and sports-betting among *boda boda* riders in an urban setting in Uganda. We hypothesized that alcohol consumption and cigarette smoking are associated with increased likelihood of sports-betting. Smoking and heavy alcohol consumption in early adulthood have long-term negative impact on health, earnings and employment later in life especially among males [18]. The data from this study will provide evidence to support measures or policies to regulate sports betting in resource limited settings where the practice is on the increase and also potentially support the integration of alcohol and sports-betting regulation.

## Methods

### Setting

We conducted a cross sectional study in Mbarara municipality located in Mbarara district in south-western Uganda. The municipality is a mixed urban and peri-urban setting with an estimated population of nearly 100,000 inhabitants. Located 250 km from the capital Kampala, it is the largest urban setting in southwestern Uganda.

### Study participants and sampling

The *boda boda* cyclists are predominantly male, with a very small number of female riders [19]. The riders are identifiable by the stage where they operate from. A stage is an area where a group of riders congregate and wait for passengers and the stages are scattered across the municipality. Each *boda boda* stage has a different number of riders ranging from a few to almost 15 in the more crowded areas of the municipality.

We used a multi stage sampling approach to select the stages from which our respondents were enrolled. In this multi-stage approach, we started at the division (or sub-county), the largest geographical unit in the municipality. We purposively selected divisions that had the largest number of *boda boda* cyclists. We then listed all the stages in the selected divisions using unique codes in an Excel sheet and then performed simple random sampling to select the *boda boda* stages. At the selected stages, we conducted a census of all *boda boda* cyclists and randomly selected from the list. We selected an equal number of cyclists from each stage. Our sample size calculation was adjusted for the design effect arising from potential clustering of riders at a given stage. We collected data using a questionnaire that was translated into the local language for those unable to speak English.

### Inclusion and exclusion criteria

The *boda boda* cyclists were eligible to participate if they were residing within Mbarara municipality, had been riding for at least 6 months, a period required for them to obtain an official registration from the Municipal commercial officers. Participants were enrolled if they provided individual informed consent to participate in the study. We enrolled participants of 18 to 59 years of age. We did not enroll those who operate on night duty as they were not available for interview during normal working hours.

### Measurements

We designed a tool (attached as supplementary material) and collected data on social demographics and income. We also collected data on sports-betting, and asked if they had ever been involved in sports-betting, and the frequency of their involvement. The participants in our study do not have access to other forms of gambling other than sports-betting, and therefore sports-betting and gambling are used interchangeably in our description. We collected data on alcohol consumption and used the Alcohol Use Disorders Identification Test (AUDIT) tool, a 10-item screening tool developed by the World Health Organization [20]. We asked participants about their smoking habits, namely whether they had ever smoked and if they had smoked in the past 6 months.

### Data analysis

We summarized numeric data using descriptive statistics namely means and standard deviations or medians with interquartile ranges (IQR), and categorical data using frequencies and percentages. We coded sports-betting as a dichotomous outcome. We considered a rider to have engaged in sports-betting if they reported they had been actively involved in sports-betting in the past 30 days. We defined active sports-betting as spending money to complete a transaction in a sports-betting activity. We coded the outcome as “1” when the participant was involved in sports-betting or “0” when not involved. Alcohol consumption was classified into three categories based on the AUDIT-C score namely low, moderate and high based on the volume of consumption with the smallest consumers in the low category and the largest consumers in the high category. An AUDIT score of 0 to 4 was considered low, 5 to 8 was classified as moderate and any score higher than 8 was considered high or hazardous alcohol use.

We examined categorical variables such as marital status, residence and alcohol consumption by sports-betting status using the Chi-square test, when the cell count was large (≥5) or the Fisher’s exact test when cell count was small (< 5). We examined continuous variables such as age by sports-betting status, and here we used the student’s t-test when the data were normally distributed or the Wilcoxon sum rank test when skewed. We tested our hypothesis using a two sided test approach and 0.05 as the level of significance.

The proportion of the outcome variable was considered highly frequent, so the odds ratio (OR) is not an appropriate measure of effect because it would overestimate the strength of the association [21, 22]. Therefore, for the regression analysis, we computed risk ratios (RR) using modified Poisson regression with robust error variance to control for potential violations of the assumptions of Poisson regression analysis. The primary exposure for this analysis was alcohol consumption and the outcome was sports-betting. In the regression analysis, we calculated unadjusted RR (uRR) and their 95% confidence intervals (CI). We then fitted a multivariable model for variables that showed *p* values less than 0.1 at the unadjusted analysis and then reported the adjusted RR (aRR) and 95% CI. Prior to the multivariate analysis, we checked for multicollinearity and considered variance inflation factor (VIF) greater than 10 as indicator of collinearity. We also checked model fitness using Akaike Information Criteria (AIC), link test, and goodness of fit (GOF) test. We considered the AIC (389.4) from our model as low and a statistically non-significant *p*-value for the link test (*p* = 0.850) and the GOF test (Pearson GOF = 317.2, chi-square = 386, *p* = 0.996) as indicative of good model fit. Furthermore, we checked for the assumption of normal distribution by plotting a graph of residuals of the fitted versus residual values.

### Human subjects

The study was approved by the Research Ethics Committee at Mbarara University of Science and Technology. Study procedures were adherent to the requirements under the Helsinki declaration. Participants were invited and voluntarily accepted to participate. Individual informed and written consent was obtained from all participants, and all were at least 18 years of age. Consent process was conducted in the local language to enhance understanding. In the reporting of results, divisions of residence have been left anonymous to protect group confidentiality.

## Results

### Socio-demographic characteristics of participants

We approached 425 eligible riders and enrolled 401 of them. Twenty four (5.6%) participants of those approached declined to participate. The mean age (standard deviation) of those enrolled participants was 29.3 ± 5.9 years (median age, 29.0; Interquartile range or IQR, 25–32). Table 1 presents a summary of the socio-demographic characteristics of participants stratified by sports-betting status. There was no statistically significant difference in mean age between participants who engaged in sports-betting and those who never: 28.9 ± 5.4 versus 29.4 ± 6.1, respectively; *p* = 0.56. Statistically significant differences by sports-betting were observed with respect to time for end of work day (*p* = 0.043), alcohol consumption (*p* = 0.002) and smoking status. Riders who end their work day before 7:00 pm are more likely to engage in sports-betting compared to those who end their work day after 7:00 pm. Alcohol consumption and smoking are more frequent among persons who were engaged in sports-betting compared to those who were not. The other variables such as the division of residence, religion, educational level, marital status, number of children, income per day and ownership of a motorcycle taxi did not differ (all *p* > 0.05) between participants who engaged in sports-betting and those that did not. Although the riders who smoke cigarettes were more likely to engage in sports-betting, use of marijuana was not related to sports-betting.

**Table 1:** Characteristics of motorcycle taxi riders by sports betting status, Mbarara municipality, Uganda

		Engaged in sports betting		
Characteristics	Level	No (*n* = 327)	Yes (*n* = 74)	*p* value
Division of residence	A	110 (33.6)	23 (31.1)	0.185
	B	79 (24.2)	24 (32.4)	
	C	108 (33.0)	17 (23.0)	
	D	30 (9.2)	10 (13.5)	
Mean age (SD)		29.42 (6.07)	28.97 (5.41)	0.558
Religion	Catholic	123 (37.6)	25 (33.8)	0.131
	Protestant	138 (42.2)	38 (51.4)	
	Moslem	45 (13.8)	4 (5.4)	
	Others	21 (6.4)	7 (9.5)	
Educational level	None/primary	204 (62.6)	44 (59.5)	0.618
	Secondary/Tertiary	122 (37.4)	30 (40.5)	
Marital status	Never married	90 (27.5)	18 (24.3)	0.428
	Married	218 (66.7)	54 (73.0)	
	Separated	19 (5.8)	2 (2.7)	
Number of children	≤2	211 (64.9)	49 (66.2)	0.977
	3–5	100 (30.8)	22 (29.7)	
	> 5	14 (4.3)	3 (4.1)	
Owns a *boda boda*	No	169 (51.8)	40 (55.6)	0.659
	Yes	157 (48.2)	32 (44.4)	
Income per day in dollars	< 5	135 (41.7)	40 (54.1)	0.071
	≥5	189 (58.3)	34 (45.9)	
End of work day	Before 7.00 pm	38 (11.8)	16 (21.6)	0.043^*^
	After 7.00 pm	283 (88.2)	58 (78.4)	
Alcohol consumption	Low	266 (81.3)	49 (66.2)	0.002^**^
	Moderate	40 (12.2)	21 (28.4)	
	High	21 (6.4)	4 (5.4)	
Ever smoked cigarettes	No	296 (90.5)	52 (70.3)	< 0.001***
	Yes	31 (9.5)	22 (29.7)	
Smoked cigarettes in the past 6 months	No	301 (92.0)	58 (78.4)	0.001***
	Yes	26 (8.0)	16 (21.6)	
Ever smoked marijuana	No	305 (93.3)	66 (89.2)	0.337
	Yes	22 (6.7)	8 (10.8)	
Ever smoked marijuana in the past 6 months	No	320 (98.8)	71 (95.9)	0.24
	Yes	4 (1.2)	3 (4.1)	

Note: 1) ^*^ *p* < 0.05, ^**^ *p* < 0.01, ^***^ *p* < 0.001 at 5% level of significance

### Frequency and pattern of sports-betting

The results for frequency and pattern of sports-betting are presented in Table 2 below. Of the 401 participants, 74 (18.5%) had engaged in sports-betting in the past 30 days. Among those that reported sports-betting in the past 30 days, 31 or 41.9% engaged less than weekly, 27 (36.5%) engaged weekly while 16 (21.6%) daily or almost daily.

**Table 2:** Frequency and pattern of sports-betting among motorcycle taxi riders in Mbarara municipality, Uganda

Characteristics	Category	n (%)
Ever engaged in sports-betting in the past 30 days (*n* = 401)	No	327 (81.5)
	Yes	74 (18.5)
Pattern of sports-betting in the past 30 days (n = 74)	Daily or Almost daily	16 (21.6)
	Weekly	27 (36.5)
	Less than weekly	31 (41.9)

### Factors associated with sports-betting

We present the results of the regression analysis in Table 3. In the unadjusted analysis, data show that earning five or more dollars per day was not significantly associated with betting (Unadjusted risk ratio or uRR, 0.67; 95% CI, 0.44–1.01), although marginally significant. Riders who end work day after 7.00 pm (uRR, 0.55; 95% CI, 0.36–0.93) were less likely to engage in sports-betting. Alcohol consumption was significantly associated with sports-betting. There was a more than 2 fold increase in the likelihood of sports-betting among those in the moderate alcohol consumption category (uRR, 2.21; 95% CI, 1.44–3.41) with the low consumption category as the referent. The association was not significant among those in the high consumption category (uRR, 1.03; 95% CI, 0.40–2.62). Smoking was significantly associated with sports-betting uRR 2.36, 95% 1.50, 3.71).

**Table 3:** Modified poisson regression analysis of the relationship between alcohol and sports-betting among motorcycle taxi riders, Mbarara municipality, Uganda

		Modified Poisson regression analysis			
Characteristics	Level	uRR	95% CI	aRR	95% CI
Income per day/dollars	< 5	1		1	
	≥5	0.67	(0.44,1.01)	0.74	(0.49,1.11)
End of work day	Before 7.00 pm	1		1	
	After 7.00 pm	0.57^*^	(0.36,0.92)	0.62^*^	(0.39,0.99)
Smoked cigarettes in last 6 months	No	1		1	
	Yes	2.36^***^	(1.50,3.71)	1.85^*^	(1.13,3.01)
Alcohol consumption	Low	1		1	
	Moderate	2.21^***^	(1.44,3.41)	1.86^**^	(1.18,2.94)
	High	1.03	(0.40,2.62)	0.97	(0.37,2.58)

Note: 1) ^*^ *p* < 0.05, ^**^ *p* < 0.01, ^***^ *p* < 0.001 at 5% level of significance; 2) 95% confidence intervals for risk ratio (RR) in brackets; 3) aRR: Adjusted Risk Ratio; 4) uRR: Unadjusted Risk Ratio; 5) Risk Ratios are exponentiated coefficients

After adjusting for potential confounders, riders who end the work day after 7.00 pm were 38% (adjusted risk ratio or aRR, 0.62; 95% CI, 0.39–0.99) less likely to engage in sports-betting compared to those who end the day before 7.00 pm. We observed an inverted U relationship between alcohol consumption and sports-betting. Riders in the moderate alcohol consumption category were nearly 2 times more likely to engage in sports-betting compared to those in the low consumption category (aRR,1.86; 95% CI, 1.18–2.94). However, among those in the high consumption category, the relationship was not significant. Smoking remained significantly associated with sports-betting after adjustment for all other variables in the model with aRR 1.85 (95% CI 1.13, 3.01). Income earned per day was not significantly related to sports-betting but those who ended their work day after 7 pm were less likely to engage in sports-betting compared to those who end their work day earlier.

## Discussion

Our data show that 18.5% of the motorcycle taxi riders have been involved in sports-betting in the most recent month, and that among those that engage in sports-betting, about one fifth of them engage daily or nearly daily. Our data show that riders who stop work earlier are more likely to engage in gambling, but most importantly, alcohol consumption and cigarette smoking were significantly associated with gambling. Income earned per day is unrelated to the likelihood to get engaged in sports-betting. Our study is one of the few from sub Saharan Africa that have examined the association between alcohol consumption and gambling.

The frequency of sports-betting in our study population is comparable to that from a study in the United States [23], although this was an American student population. However, the frequency in our study population is only half of that demonstrated in a South Africa population [24]. This study in South Africa was conducted among a general adult population, mostly low income poor population. Our study population consists of low income earners as well but younger. However, the frequency gambling in South Africa is much higher and may be because gambling is well established there and has been practiced since the 1990s and with a variety of options unlike in Uganda. A Ugandan study showed that about one in every four adults in the capital Kampala, had engaged in some form of gambling in the past 12 months [6], which is comparable to the frequency of gambling in our study. Studies elsewhere in sub Saharan Africa show gambling is prevalent in both rural and urban settings mostly among the youth as pass time and some have sold household goods to finance their gambling activities [25]. In our study population, sports-betting was the only form of gambling available to the riders. Other forms of gambling such as casinos are available in the capital Kampala, but are likely to be accessed by the higher income earners as the costs of gambling in the casinos are likely to be higher.

Our findings of the association between alcohol consumption and gambling are in agreement with those of a study in South Africa among a population of gamblers [26]. Our findings are also consistent with those of a study conducted among residents of Kampala city, Uganda [5]. The results confirm our primary hypothesis and agree with several other studies outside the African continent such as those studies conducted in the United States [27, 28], United Kingdom [29], and Sweden [30]. We observed an inverted U association, an indication that among the heavy drinkers, there was no association with sports-betting. It is not clear whether alcohol consumption precedes gambling or vice-versa, in the pathway and whether there is a causal relationship and what the mechanism may be. Studies suggest alcohol drinking may increase the propensity to gamble [31]. There is possibility that heavy drinkers lose their income to drinking and have limited resources left to gamble, explaining the inverted U shape relationship.

Our study also shows a significant association between cigarette smoking and sports-betting. This finding fits our hypothesis and also agrees with findings from other studies [32]. Alcohol and nicotine in the cigarettes are both substances of addiction and their close association with gambling, which is also addictive is not unexpected. Our data adds to the limited existing evidence of the link between the use of alcohol and cigarettes with gambling in sub Saharan Africa [26]. For the continent, these results are significant because of the close association of all these exposures with mental health conditions, which are much neglected area on the continent. Alcohol, substance abuse, anxiety and depression are highly prevalent among persons with problem gambling [33]. Marijuana was not commonly used and our data did not have sufficient numbers to explore its association with sports-betting.

The finding that participants who ended work after 7.00 pm are less likely to engage in sports betting is novel but not surprising. We hypothesized that the riders who leave work before 7.00 pm have more time to watch football matches on television, which are usually played in the late evening hours and to participate in sports betting. The riders who end work after 7.00 pm would have less opportunity to follow live games and place their bets.

It is important to note that we conducted our study among motor cycle taxi riders, a sub-group of the population that comprises predominantly young male Ugandans. We chose this group because they represent a large proportion of urban male youth who earn a living from a meager daily income. Youth unemployment is very high in Uganda with more than 60% of youth aged between 18 and 35 years facing unemployment. Motorcycle taxis provide an option for them to earn a living. These results are therefore generalizable to a large proportion of out of school male youth and young adults in Uganda.

### Study strengths and limitations

Although our study provides unique data from sub Saharan Africa, it has some weaknesses. We did not measure the severity of the gambling. There are well established tools to do this such as the Gambling severity index score. Future studies should consider including the measurement of the grade of severity of gambling and the social and economic consequences of gambling. Our sample size was relatively small and may limit the external validity of our findings. In addition, we had a 5.6% non-response, and although this is small, we did not compare the features of these non-responders to those who were enrolled and there might have been a bias introduced. Third, we did not collect data on amounts of money spent on alcohol consumption, smoking, and sports-betting, and to establish the impact of expenditures on these items on each other. These gaps should be the focus for further research. Furthermore, qualitative data are needed to gain in-depth understanding on the reasons or facilitators of sports betting and the subsequent consequences.

The strength of our study is that it presents data on gambling, a practice that is growing in sub Saharan Africa, yet under researched on the continent. The finding of an inverted U relationship between alcohol consumption and sports-betting is novel and should be further examined and replicated in other studies. Although several studies have shown the association between substance abuse and gambling [23, 34, 35], very few such studies have been done in resource limited settings.

## Conclusions

In conclusion, our study has shown that gambling via sports-betting occurs in almost two in every 10 *boda boda* motorcyclists, a low income category group of predominantly male youth and young adults in Uganda. Our study shows that sports-betting is associated with alcohol consumption and cigarette smoking and is also more likely to occur among riders who end the work day earlier. Although our data do not provide evidence on the long term outcomes of sports betting, there is concern that sports-betting is on the rise and some of the participants bet daily or nearly daily and may be at risk for developing problem gambling and addiction. We recommend that future studies should examine this relationship.

Our findings have important implications. There is a need to put in place policies and structures to regulate the use of sports-betting services. The regulation will likely face significant challenges because sports-betting services can be accessed easily via mobile phones by users in the privacy of their homes. In addition, sports-betting is legal in Uganda and many other countries in sub Saharan Africa and the industry is lucrative and contributes revenue to the government coffers through taxation. There is a likelihood that interventions to regulate access to sports-betting may be met with resistance from certain stakeholders that benefit from the business. We recommend that future research should explore gambling expenditure. Also, research on policy should be done to address the current regulatory frameworks, industry influence on government and regulation, advertising and marketing to fully contextualize any proposed interventions. Lastly, given the close association between sports-betting and alcohol consumption, interventions should be co-packaged to deal with the two public health challenges concurrently.

## Supplementary Information

**Additional file 1.** Supplementary material-study tool (Study tool).

## Data Availability

Data are available upon reasonable request from corresponding author (fbaj@yahoo.com) and with approval from the Research Ethics committee. Please email sec.rec@must.ac.ug

## Abbreviations

AUDIT: Alcohol Use Disorders Identification Test
aRR: Adjusted relative risk
GOF: Goodness of fit
uRR: Unadjusted relative risk
