# Supplementary material for: Alcohol consumption and sports-betting among young male motorcycle taxi boda boda riders in urban southwestern Uganda
Source: BMC Public Health. 2021 Feb 16;21:363. doi: 10.1186/s12889-021-10406-7 (PMC7885426; doi:10.1186/s12889-021-10406-7)
Supplement: Supplementary file 1 — Additional file 1. Supplementary material-study tool (Study tool). [file 12889_2021_10406_MOESM1_ESM.pdf]

QUESTIONNAIRE

A. DEMOGRAPHIC QUESTIONS

Serial number……………………………………… Starting time……………………

1. Location/Division…………………………… 2. Age (completed years) ……

3. What is your religion?
   1. Catholic
   2. Protestant
   3. Moslem
   4. Others

4. How many minutes do you use to ride from home to the town center ……………………………

5. What is your education level?
   1. No formal education
   2. Primary
   3. Secondary
   4. Tertiary/university/higher

6. What is your current marital status?
   1. Never married
   2. Currently married/Cohabiting
   3. Divorced/Separated
   4. Others

7. Estimated income per day ....Ug shillings

8. How many children do you have? ………………………….

9. Whom do you live with?
   1. Live with parents
   2. Live away from parents

10. Have you ever participated in sports betting?
    1. Yes
    2. No if no, skip to B below

11. Have you spent money to engage in sports betting in the last 30 days?
    1. Yes
    2. No

12. How often do you do you sports bet?
    1. Never
    2. Less than monthly
    3. Monthly
    4. Weekly
    5. Daily or almost daily

13. How much money do you spend on sports betting in a week? ___________ Ug shs

B. SOCIAL SUPPORT

Question

1. Is there someone who can cheer you up when you are sad?
   1. Yes
   2. No

2. Do you have people who offer you help in case you need it?
   1. Yes
   2. No

3. Do you have people you can reach out to when you need advice
   1. Yes
   2. No

4. Do you have someone who can comfort you when you need
   1. Yes

it?	2. No
5. Do you have a hard time keeping pace with your friends?	1. Yes<br>2. No
6. If you were stranded 10 miles from home, is there someone you could call who would come and get you?	1. Yes<br>2. No
7. Are most of your friends are more interesting than you are?	1. Yes<br>2. No
8. Is there is someone who takes pride in your accomplishment?	1. Yes<br>2. No
9. When you feel lonely, are there people you can talk to?	1. Yes<br>2. No
10. Do you often meet or talk with family and friends?	1. Yes<br>2. No

C. **RISKY SEXUAL BEHAVIOR**

**Question**	**Answers**
1. Have you ever had sexual intercourse?	1. Yes<br>2. No
2. The last time you had sexual intercourse, was it protected?	1. Yes<br>2. No
3.The last time you had sexual intercourse was with	1. Regular partner<br>2. casual partner
4. How many sexual partners have you had in the last 6 months?	……………………………..
5. How many sexual partners have you had in the last 12 months?	………………………….
6.How many sexual partners have you had in your lifetime	…………………………………
7. Have you ever had sex under the influence of alcohol?	1. Yes<br>2. No
8. Have you ever had sex in exchange for money or favors?	1. Yes<br>2. No
9. Have you ever had sex with a commercial sexual workers/prostitute?	1. Yes<br>2. No
10. Have you ever been paid to have sexual intercourse?	1. Yes<br>2. No
11. Have you ever exchanged rides for sex?	1. Yes<br>2. No

12.How frequently have you used condoms when having sex with your regular partner(s)	1. All the time<br>2. Most of the time<br>3. Some of the time<br>4. Never
13. How frequently have you used condoms when having sex with your casual partners?	1. All the time<br>2. Most of the times<br>3. Some of the time<br>4. Never
14.How often have you used a condom when you have been paid for sex in the last month	1. All the time<br>2. Most of the time<br>3. Some of the time<br>4. Never

STDs

15. Have you ever suffered from an STD?	1. Yes<br>2. No
16.Have you ever been told by health worker that you have STD?	1. Yes<br>2. No
17. Have you ever had an ulcer/wound on your private parts?	1. Yes<br>2. No
18. Have you ever passed pus in your urine?	1. Yes<br>2. No
19. Have you ever had pain in passing urine?	1. Yes<br>2. No

D. ALCOHOL USE

Read the questions as written, record answers carefully, now I am going to ask you some questions about your use of alcoholic beverages during the past year. The alcoholic beverages mean local beer, wine, vodka etc. the answers will be coded in terms of standard drinks. Place the correct answer number in the box at the right

1. How often do you have a drink containing alcohol
(0) Never **(skip to Qs 9-10)**
(1) monthly or less
(2) 2 to 4 times a month
(3) 2to 3 times a week
(4) 4 or more times a week

2. How many drinks containing alcohol do you have on a typical day when you are drinking
(0) 1 or 2
(1) 3 or 4
(2) 5 or 6
(3) 7,8 or 9
(4) 10 or more

3. How often do you have six or more drinks on one occasion?
(0) Never
(1) less than a monthly
(2) monthly
(3) weekly
(4) daily or almost daily

Skip to question 9-10 if total score for questions 2 and 3 =0

4. How often during the last year have you found that were not able to stop drinking once you had started
(0) Never
(1) less than monthly
(2) monthly
(3) weekly
(4) daily or almost daily

5. How often during the last year have you failed to do what was normally expected from you because of drinking
(0) Never
(1) less than monthly
(2) monthly

6. How often during the last year have you needed a first drink in the morning to get yourself going after a heavy drinking session?
(0) Never
(1) less than monthly
(2) monthly
(3) weekly
(4)Daily or almost daily

7. How often during the last year have you had a feeling of guilt or remorse after drinking?
(0) Never
(1) less than monthly
(2) monthly
(3) weekly
(3) daily or almost daily

8. How often during the last year have you been unable to remember what happened the night before because you had been drinking?
(0) Never
(1) less than monthly
(2) monthly
(3) weekly
(4) daily or almost daily

9. Have you or someone else been injured as a result of your drinking?
(0) No
(2) yes but not in the last year
(4) yes during the last year

10. Has a relative or friend or a doctor or another health worker been concerned about your drinking or suggested you cut down
(0) No
(2) yes but not in the last year
(4) yes during the last year

(3) weekly
(4) daily or almost daily

Record total of specific items here
